# Age-related increase in amyloid plaque burden is associated with impairment in conditioned fear memory in CRND8 mouse model of amyloidosis

**DOI:** 10.1186/alzrt124

**Published:** 2012-06-14

**Authors:** Amanda Hanna, Kayleigh Iremonger, Pritam Das, Dennis Dickson, Todd Golde, Christopher Janus

**Affiliations:** 1 Center for Translational Research in Neurodegenerative Disease and Department of Neuroscience, University of Florida, 1275 Center Dr., Gainesville, FL, 32610, USA; 2 Department of Neuroscience, Mayo Clinic, 4500 San Pablo Rd., Jacksonville, FL, 32224, USA; 3 Neurobiology Division, The Roslin Institute and R(D)SVS, University of Edinburgh, Easter Bush, Midlothian, EH25 9RG, UK

## Abstract

**Introduction:**

The current pathological confirmation of the diagnosis of Alzheimer's disease (AD) is still based on postmortem identification of parenchymal amyloid beta (Aβ) plaques, intra-neuronal neurofibrillary tangles, and neuronal loss. The memory deficits that are present in the early stages of AD are linked to the dysfunction of structures in the entorhinal cortex and limbic system, especially the hippocampus and amygdala. Using the CRND8 transgenic mouse model of amyloidosis, which over-expresses a mutant human amyloid precursor protein (*APP*) gene, we evaluated hippocampus-dependent contextual and amygdala-dependent tone fear conditioned (FC) memory, and investigated the relationship between the fear memory indices and Aβ plaque burden.

**Methods:**

Mice were tested at three, six, and 12 months of age, which corresponds to early, mild, and severe Aβ plaque deposition, following a cross-sectional experimental design. We used a delay version of the fear conditioning paradigm in which tone stimulus was co-terminated with foot-shocks during exploration of the training chamber. The Aβ plaque burden was evaluated at each age after the completion of the behavioral tests.

**Results:**

CRDN8 mice showed context fear memory comparable to control mice at three and six months, but were significantly impaired at 12 months of age. In contrast, the tone fear memory was significantly impaired in the model at each age of testing. The Aβ plaque burden significantly increased with age, and was correlated with the overall impairment in context and tone fear memory in the CRND8 mice within the studied age.

**Conclusions:**

Our data extend previous studies showing that other APP mouse models exhibit impairment in fear conditioned memory, by demonstrating that this impairment is progressive and correlates well with an overall increase in Aβ burden. Also, the demonstrated greater sensitivity of the tone conditioning test in the identification of age dependent differences between CRND8 and control mice suggests that this paradigm might be particularly suitable in studies evaluating potential therapeutics related to memory improvement in mouse models of amyloidosis.

## Introduction

Alzheimer's disease (AD) is the leading cause of dementia in the elderly, affecting more than 35 million people worldwide [1]. Currently, confirmation of a clinical diagnosis of AD still requires post mortem identification of parenchymal amyloid beta (Aβ) deposits and intra-neuronal neurofibrillary tangles composed of abnormally phosphorylated tau protein [2-5] and severe loss of brain tissue [6-8]. In the near future, cerebrospinal fluid (CSF) measures of Aβ and tau or amyloid imaging may be utilized to provide pre-mortem confirmation of the AD diagnosis. Senile amyloid plaques are found in large numbers in the limbic system, including amygdala (AD is often referred to as 'limbic dementia' [9]), hippocampus, and associative cortices which are affected first during the disease progression [10-18].

Transgenic mice, over-expressing the mutated human amyloid precursor protein (*APP*) gene, provide a valuable tool for investigating the associations between amyloidosis, neuronal dysfunction, and cognitive impairment [19-23]. In the present study, we investigated the age-progressing Aβ plaque burden and corresponding changes in conditioned fear memory in a transgenic mouse model, denoted CRND8. Previous characterizations of this model revealed impairments in spatial reference [24-26] and spatial working [27] memory, and in associative learning of conditioned taste aversion [28]. Other abnormalities reported in CRND8 mice included increased stereotypic behavior [29], brain inflammation [30] and increased sensitivity to experimentally induced seizures [31].

In our study, we adopted a delay fear conditioning (FC) training paradigm in which an initially neutral conditioned stimulus (CS), usually a tone, is simultaneously presented or co-terminates with an unconditioned stimulus (US), typically a foot-shock [32,33]. Following the CS-US pairing(s), mice display an anti-predatory freezing response both in the presence of a salient CS (tone conditioned fear memory) or when being placed in the original training chamber in which they experienced the US (contextual fear conditioning memory). It has been shown that the contextual fear memory depends on an intact hippocampus [34,35], while the cued fear memory depends on an intact amygdala [36,37].

The aim of the present study was to evaluate the contextual and cued fear memory of CRND8 mice at the age of three, six, and 12 months, which corresponded to the onset of low, moderate, and severe Aβ plaque deposition in the brain of these mice [38], and to associate the Aβ plaque burden with the context and tone memory indices. The results demonstrated that the Aβ plaque burden significantly increased within the studied age range, and it was significantly associated with an overall impairment in contextual and tone fear memory in CRND8 mice. The oldest, 12 month-old, CRND8 mice showed impairment in both types of memory. While the context memory of the younger, three and six month-old, CRND8 mice was comparable to control littermates, the tone fear memory of the CRND8 mice was significantly impaired at each age of testing. The apparent increase in the sensitivity of the detection of age-dependent onset of memory impairment using tone fear conditioning makes this test an attractive potential diagnostic tool during evaluation of the efficacy of potential therapeutics on memory function in the CRND8 mouse model.

## Materials and methods

### Mice

The transgenic CRND8 mice over-express mutant forms of human *APP *genes (*Swedish; KM670/671NL *+ *Indiana; V717F*) [26,38] implicated in AD [39,40]. This model shows rapid onset of extra-cellular Aβ deposits at 2.5 to 3 months of age, with coinciding impairment in spatial reference memory [26]. Dense-core Aβ plaques and neuritic pathology appear at five months [38].

Three cohorts of transgenic (Tg) CRND8 and non-transgenic (nTg) littermates (hybrid genetic background, C57BL/6//C3H) at ages three (N = 27, 13/14 Tg/nTg), six (N = 28, 11/17 Tg/nTg), and 12 (N = 24, 11/13 Tg/nTg) months were used. The physical condition and sensorimotor propensities of the CRND8 mice did not differ from their control nTg littermates within the studied age range as evaluated in the SHIRPA (**S**mithKline Beecham Pharmaceuticals; **H**arwell, MRC Mouse Genome Centre and Mammalian Genetics Unit; **I**mperial College School of Medicine at St Mary's; **R**oyal London Hospital, St Bartholomew's and the Royal London School of Medicine; **P**henotype **A**ssessment) phenotyping screen (data not shown). The cohorts within each genotype were female biased (median for males = 3.5, for females = 9). The mice were genotyped at weaning by analysis of tail DNA with a human APP hybridization probe, as described previously [38]. They were housed in same-sex groups of two to four under standard laboratory conditions (12:12 hours light/dark cycle, lights on at 0600 hours) with a room temperature of 21°C, and water and food available *ad libitum*. All tests were performed during the light phase between 09:00 and 14:00 hours. All procedures were approved by the Institutional Animal Care and Use Committee of Mayo Clinic Jacksonville and are in accordance with Association for Assessment and Accreditation of Laboratory Animal Care International (AAALAC) and the National Institutes of Health Guide for the Care and Use of Laboratory Animals guidelines.

### Primary neurological and sensorimotor examination

The SHIRPA protocol [41,42] involves a series of tests assessing the physical condition of the mice. The following phenotypes are measured: (1) body position in a cage, respiration, tremor, transfer arousal, palpebral closure, piloerection, (2) reflexes - touch escape, pinna reflex, trunk curl, limb grasping, visual placing, negative geotaxis and righting reflex, and (3) grip strength. The screen takes altogether about five to seven minutes per mouse.

### Fear conditioning test

The conditioning procedure was carried out in four identical chambers (25.3L × 29.5W × 29.5H cm; Coulbourn Instruments.). The total floor area of each chamber was 746 cm^2^. The chambers were constructed from aluminum (sidewalls and ceiling) and Plexiglas (rear and front walls). They were placed individually in sound-attenuated cabinets with black inside walls (interior dimensions: 43.3L × 55.3W × 58.5H cm; Coulbourn Instruments.), which were located in a dedicated room. A ventilation fan in each cabinet provided 50 dB of background noise, and a 24V DC white light, mounted on a wall of each chamber, provided illumination (65 lux at the floor level). A speaker mounted in the wall opposite to the light delivered an acoustic CS. The floor of each chamber, which consisted of 26 stainless steel rods (3 mm in diameter) spaced 11 mm center to center, was wired to a precision-regulated shocker (H13-15, Coulbourn Instruments). A camera mounted above the chamber recorded mouse activity. Conditioning was assessed by the analysis of the fear response expressed as freezing behavior with the aid of the FreezeFrame program (v. 3.06, Actimetrics). Freezing was defined as the cessation of all movements other than respiratory activity [43].

### Conditioning procedure and memory tests

Mice were exposed to the context of a training chamber and a tone, both initially novel and neutral stimuli, in one training session. They were transported in squads of four in individual containers filled with home cage bedding and placed singly in the conditioning chamber. During training, the mice received two pairings between a tone (80 dB, pulse (six clicks per second (c.p.s)), 30 seconds duration) and a 0.45 mA foot shock (two seconds duration, co-terminated with a tone). The first CS-US pairing was delivered at the end of 120 seconds of the initial exploration of the chamber, and the second following a 60-second interval. After the second CS-US pairing the mice were given a 60-second post-training period. The total duration of the training session was 300 seconds. After a day of recovery, the mice were returned to their respective conditioning chambers and tested for fear-induced freezing to the context in which they received foot shocks. The test, carried out in an extinction mode with no shock administered, lasted 300 seconds. The following day, the mice were tested for the association between the tone and the foot-shock in a modified chamber. The floor and the walls of the chamber were replaced with plastic inserts (opaque white for the floor, and semi-transparent white at the front and opaque green at the back for the walls), which also eliminated corners in the chamber. The total floor area of the modified chamber was about 671 cm^2^. A Petri dish containing a drop of a Pure Lemon Extract (McCormick) was placed underneath the floor of each chamber to provide a distinct novel odor in the chamber. The above modifications did not change the light intensity in the chamber. The tone test lasted 360 seconds. During the first 180 seconds the mice were allowed to explore the new environment and during the second 180 seconds a tone, with the same characteristics as the tone used during training, was delivered. Mice activity was recorded during all tests.

### Quantification of Aβ deposition

At the end of the experiment, the mice were sacrificed and their brains were removed. One hemi-brain was submerged in 10% neutrally buffered formalin for immunohistochemical analysis of Aβ plaque burden. The remaining hemisphere of the brain was snap frozen and stored at -80°C for further analysis. Paraffin, coronal 5 μm sections were affixed to Fisher brand Superfrost/Plus slides to ensure adhesion. Brain sections (10 to 12 sections/set) cut at 30 μm intervals within the range of -1.22 mm to -3.08 mm from the bregma [44], including the hippocampus and amygdala, were used for analyses. All slides were deparaffinized and immunostained with the pan Aβ 1 to 16 (33.1.1) antibody (dilution 1:5000) to visualize both diffuse and core Aβ deposits. A separate set of slides was stained by anti- Aβ40 (MM32-13.1.1) antibody (dilution 1:2000) in order to selectively quantify core Aβ deposits. Stained sections were scanned with a high resolution, whole slide imaging system (0.46 μm/pixel with 20X objective lens, ScanScope™ XT, Aperio Technologies, Inc. Vista, CA, USA). The images were viewed in an ImageScope™ viewer (v. 10) and the Aβ-positive staining was detected using an automated image analysis system by applying a color deconvolution method [45] within the Hue, Saturation, Intensity (HSI) model (Color Deconvolution algorithm, Aperio Technologies, Inc., settings: hue value and width = 0.1 and 0.3, respectively, and saturation threshold = 0.04). The area of the brain including the cortex, hippocampus, and amydgala were outlined according to the mouse atlas [44], and the Aβ burden was expressed as the percent of outlined area stained positively for Aβ. Background staining was determined in the area of basal ganglia, which was devoid of Aβ-positive staining, and was set to a pixel value of 40.

### Data and statistical analyses

Since the experimental design included two between subjects factors: genotype and age, we followed two *a priori *identified approaches to data analysis. In the first, we compared the conditioned fear memory between the genotypes within the tested age range, followed by *post-hoc *analysis at each age. These analyses provided answers to age-related differences in memory scores between transgenic and control mice. The second analytical approach focused on the age-related changes in context and tone fear memory within each genotype. While the cross-sectional design of the study did not eliminate between subjects variability in the evaluation of the age-related changes within each genotype, thus decreasing slightly the sensitivity of the study, it allowed us to evaluate the Aβ pathology at each testing age and relate it to the obtained memory scores. Due to significantly female-biased groups, the analysis of possible sex effects was not performed. The overall analysis of the experiment was done by a factorial analysis of variance (ANOVA) with genotype and age as between subject factors. Where appropriate, simple effects were evaluated using one-way ANOVA. In analyses requiring multiple comparisons between means, the Bonferroni adjustment of α level minimizing Type I (family-wise) error rate was used [46]. *A priori *comparisons were performed using the Bonferroni *t *test (MODLSD), and *post-hoc *multiple pair-wise comparisons were done using the Student-Newman-Keuls (SNK) test [46]. All statistical analyses were done using the Statistical Package for Social Sciences (SPSS Inc. Chicago) version 19 for Macintosh. Comparisons between two independent groups were done using a Student t-test. Spearman's rank correlation was used to assess the associations between Aβ burden and freezing behavior, and partial correlation was used to evaluate associations while controlling for the effect of genotype. Due to the nonparametric nature of the data obtained in the SHIRPA screen, these data were analyzed using the χ^2 ^test [47]. The critical α level was set to 0.05 in all analyses. All values in the text and figures represent means ± the standard error of the mean (SEM).

## Results

### Training: exploration and response to foot-shock

There was no difference between the CRND8 and control nTg mice in the exploratory activity preceding the first CS-US presentation (data not shown). All mice spent, on average, less than 1% of the time on spontaneous pauses during the120 second exploration. Overall, older mice paused longer (F(2,73) = 4.1, *P *< 0.05), mainly due to longer breaks in motor activity of 12-month-old CRND8 mice (F(2,73) = 5.8, *P *< 0.01, genotype by age interaction). Twelve-month-old CRND8 mice spent 2.6% ± 0.9 of the time immobile, which was significantly longer than their younger counterparts (*P *< 0.01 and *P *< 0.05 for the comparisons with three- and six-month-old mice, respectively, Bonferroni t-test), but this amounted only to about three seconds of immobility during exploration. There was no difference in activity between the age cohorts of nTg mice.

**Figure 1:**
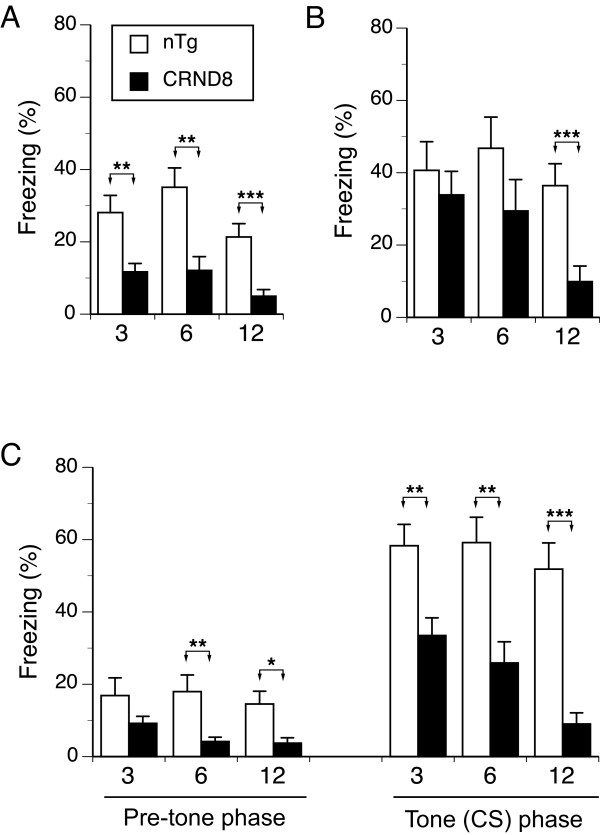
**Mean (± SEM) percent of freezing by CRND8 mice and their non-transgenic (nTg) littermates at three, six, and 12 months of age**. (**A**) CRND8 mice showed significantly lower rates of freezing as compared to nTg mice immediately following the presentation of a foot shock during training (*P *< 0.001 - genotype effect). (**B**) Overall, the context memory of CRND8 mice was impaired as compared to control nTg mice (*P *< 0.01 - genotype effect). No differences in context memory were found between nTg and Tg mice at three and six months, but at 12-months the CRND8 mice were significantly impaired (post-hoc Bonferroni t-test). (**C**) CRND8 mice froze significantly less than nTg mice during the pre-tone (left panel) and the tone (right panel) phases of the test. The CRND8 mice showed significant impairment in tone fear memory at each age of testing (right panel). Three, six, 12 on the abscissae refer to the age of testing. * *P *< 0.02, ** *P *< 0.01, *** *P *< 0.001.

The immediate freezing response to foot-shock was significantly lower in CRND8 mice than in nTg littermates (F(1,73) = 29.1, *P *< 0.001, genotype effect, Figure 1A). Also, the oldest mice of both genotypes tended to show less immediate freezing than younger mice (F(2,73) = 3.0, *P *= 0.054, age effect, Figure 1A). The examination of the effect of age on immediate freezing within each genotype revealed no significant trends in the decrease of immediate freezing in nTg or CRND8 mice (F(1,41) = 2.1, NS and F(1,32) = 2.1, NS, respectively, ANOVA simple effects), confirming a weak effect of age on immediate freezing.

### Context fear memory

The CRND8 mice showed a significantly lower freezing response during the context test than nTg littermates (F(1,72) = 7.3, *P *< 0.01, genotype effect, Figure 1B). Overall older mice showed weaker context memory (F(2,31) = 3.8, *P *< 0.05, age effect). *Post-hoc *comparisons revealed that 12-month-old CRND8 mice froze significantly less than their nTg littermates (t(22) = 3.4, *P *< 0.01); however, the contextual memory of three- and six-month-old CRND8 and nTg mice was comparable. The freezing rate of the mice during the context test was not significantly associated with the duration of pauses during initial exploration of the training chamber (r^2^(74) = 0.02, NS). The analysis of age-related changes in contextual fear memory within each genotype revealed a significant decrease in freezing to training context in CRND8 mice (F(1,32) = 3.7, *P *< 0.05, ANOVA simple effects). *Post-hoc *comparisons demonstrated that 12-month-old CRND8 mice showed a significantly lower context memory than three-month-old mice (*P *< 0.05, Bonferroni t-test), but not than six-month-old counterparts. The changes in context memory of nTg control mice due to age were not significant (F(1,41) = 0.4, NS, ANOVA, simple effects).

### Tone fear memory

The average percent of freezing time displayed by mice during the tone fear conditioning test is presented in Figure 1C. Overall, CRND8 mice froze less during the whole test than nTg mice (F(1,73) = 36.2, *P *< 0.001, genotype effect). Also, all mice froze longer during the presentation of the tone (F(1,73) = 208.2, *P *< 0.001, tone effect); however the level of freezing to tone depended on genotype (F(1,73) = 33.4, *P *< 0.001, genotype × tone interaction) and age (F(2,73) = 3.3, *P *< 0.05, age × tone interaction).

The *post-hoc *analysis revealed that CRND8 mice froze significantly less during the exploration of the altered training chamber than nTg mice (F(1,73) = 12.6, *P *< 0.001, genotype effect, Figure 1C left panel). The six- and 12-month-old CRND8 mice froze less than their three-month-old counterparts (*P *= 0.1 and *P *= 0.07, respectively, Bonferroni t-test). The freezing rate of three-month-old CRND8 mice was comparable to the freezing rate of nTg mice, which showed comparable exploration of altered context at all ages.

Overall, tone fear memory of CRND8 mice was impaired (F(1,73) = 43.9, *P *< 0.001, Figure 1C right panel, genotype effect), and was weaker in older mice (F(2,73) = 3.3, *P *< 0.05, age effect). *Post-hoc *analysis revealed that CRND8 mice showed a weaker memory than their nTg controls at each age of testing (t(25) = 3.2, *P *< 0.01, t(26) = 3.4, *P *< 0.01, and t(16) = 5.4, *P *< 0.001, for three-, six-, and 12 month-old age cohorts, respectively, Figure 1C, right panel). Within-genotypes comparisons revealed that the tone memory of CRND8 mice decreased with age (F(2, 32) = 5.7, *P *< 0.01, ANOVA, simple effects), mainly due to lower freezing in 12-month-old mice (*P *< 0.01 and *P *< 0.05 for the comparison with three- and six-month-old counterparts, Bonferroni t-test). Tone fear memory of nTg mice was not affected by age (F(1,41) = 0.3, NS, ANOVA, simple effects).

Of interest is that the significant dissociation between age-dependent onset of the impairment in the context and tone memory was caused by stronger tone memory of nTg mice as compared to the strength of their context memory at each age (t(13) = -2.8, *P *< 0.02; t(16) = -2.4, *P *< 0.05; t(12) = -2.7, *P *< 0.02, for three, six and 12 month tests, respectively). The tone and context memory of CRND8 mice were comparable (Figure 1B and 1C right panel).

### Aβ plaque burden increases with age in CRND8 mice

We previously demonstrated that amyloid plaque burden was significantly correlated with sodium dodecyl sulfate (SDS-) soluble and formic acid (FA-) extractable Aβ fractions in the CRND8 model, and that both biochemical and histo-pathological analyses of Aβ led to the same interpretations of cognitive impairment in multiple memory systems [24].

**Figure 2:**
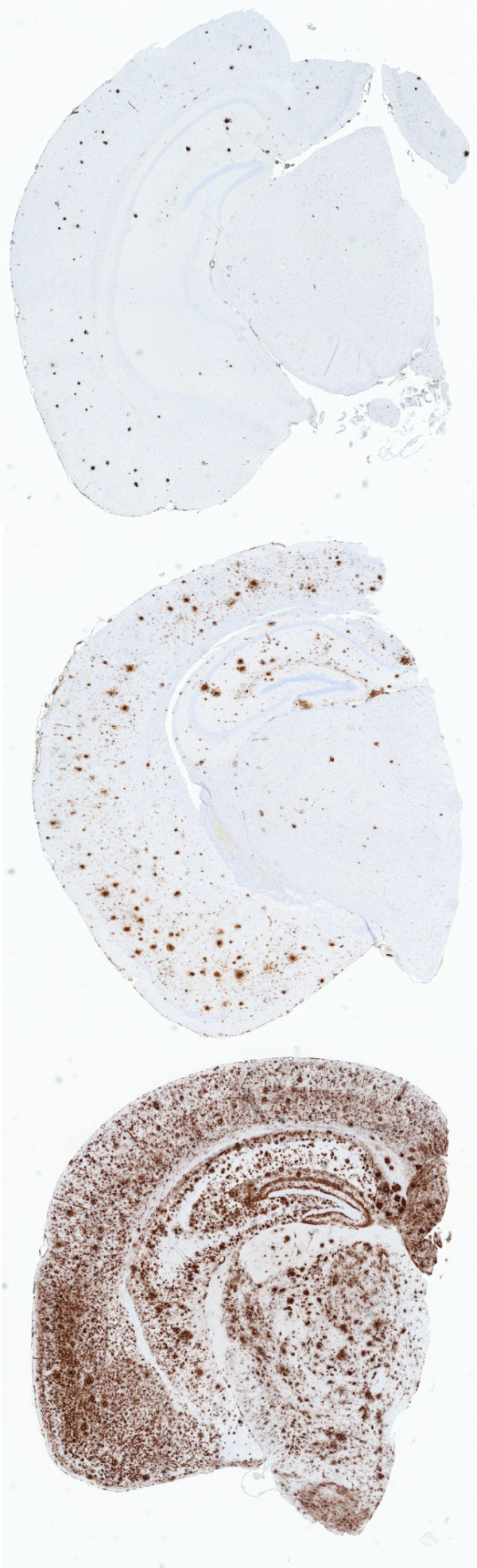
**Representative images of Aβ deposits, stained with pan Aβ 1-16 (33.1.1) antibody, in the brain sections of (A) three-, (B) six-, and (C) 12-month-old CRND8 mice**. The total amyloid burden in the combined areas of cortex, hippocampus and amygdala was 11.0%, 52.9%, and 83.1%, respectively for the sections presented in A, B, C panels.

**Figure 3:**
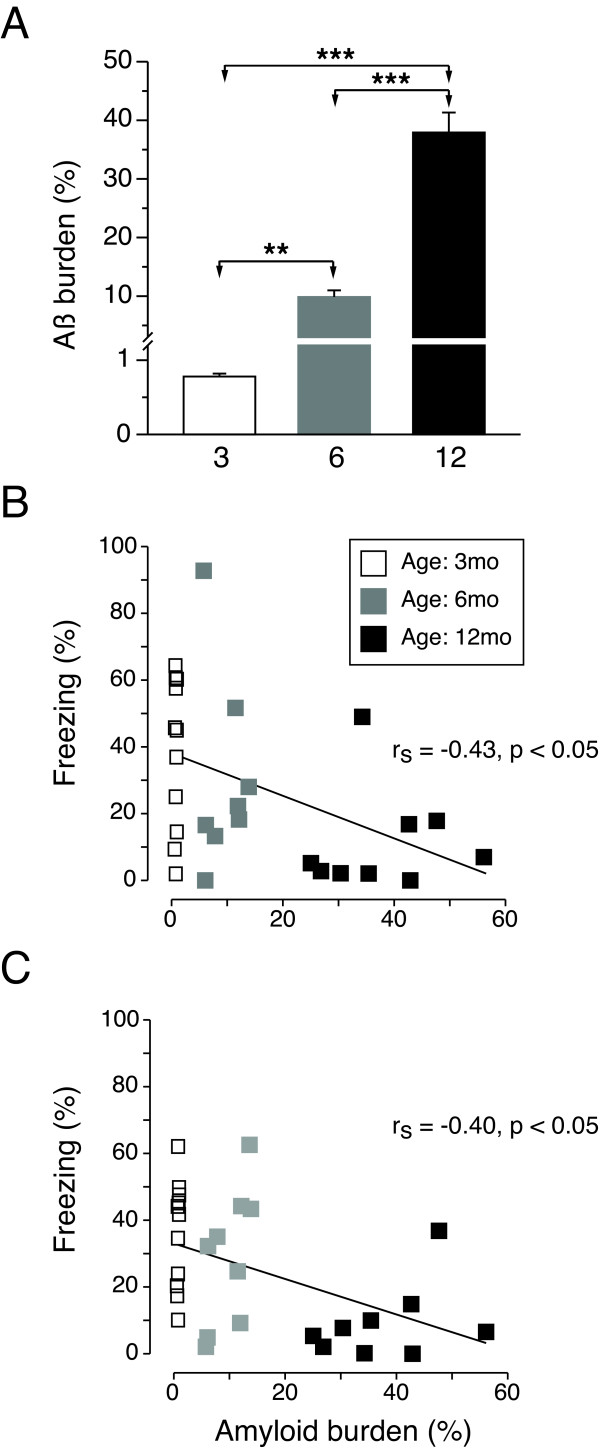
**Amyloid-β plaque burden and conditioned fear memory in CRND8 mice**. (**A**) The levels of amyloid-β burden (mean (%) ± SEM) significantly increased in CRND8 mice between the ages of three and 12 months. The progressing with age Aβ plaque burden was significantly associated with deterioration in (**B**) contextual (*P *< 0.05) and in (**C**) tone (*P *< 0.05) fear memory in CRND8 mice. ** *P *< 0.01, *** *P *< 0.001.

The representative pictures of the Aβ plaque burden in the brain of three-, six-, and 12-month-old CRND8 mice are shown in Figure 2. The Aβ plague burden increased with age (r_*S *_= 0.94, *P *< 0.001), differentiating the age cohorts of CRND8 mice (F(2,26) = 100.6, *P *< 0.001, Figure 3). *Post-hoc *comparisons revealed differences in Aβ burden between all tested age groups (3 < 6 < 12, *Ps *< 0.01, Bonferroni t-test, Figure 3A). Aβ burden at younger ages was most prominent in the cortical, hippocampal, and amygdala regions (Figure 2AB); at 12 months the Aβ deposits were observed in the whole brain, including thalamic, hypothalamic and caudate/amygdala regions (Figure 2C). We found a strong positive correlation between the Aβ plaque burden evaluated by staining with pan Aβ 1-16 antibody and the total number of Aβ dense core deposits stained by anti- Aβ40 antibody (r_*S *_= 0.9, *P *< 0.001). Consequently, the dense-core Aβ burden is not reported.

### Increase in Aβ plaque burden impairs context and tone fear memory

The increased-with-age levels of Aβ plaque burden were correlated with the impairment in context and tone fear memory in CRBD8 mice (r_*S *_= -0.43, P < 0.05 for context (Figure 3B), and r_*S *_= -0.40, *P *< 0.05 for tone (Figure 3C) memory). No association was found between Aβ plaque burden and immediate freezing following foot-shock or freezing during the pre-tone phase of the tone test.

Next, we investigated whether the variability in the Aβ plaque burden at each age of testing also reflects inverse association with context and tone fear memory. We found that variability in Aβ plaque burden (expressed by the coefficient of variation (CV)) increased with age, from 17% at three months to 34%, and 27% at six and 12 months, respectively. Moreover, the variability in memory scores of CRND8 mice differed from the variability in memory of nTg littermates. While the variance in memory scores of nTg mice was low and comparable across age groups (73%, 76%, 60%, and 38%, 49%, 50% for three, six, and 12 month context and tone memory, respectively), the variability in memory scores of CRND8 mice was higher, reaching high variance (CV > 100%) at the age of 12 months (70%, 93%, 144%, and 53%, 75%, 113% for three, six, and 12 month context and tone memory, respectively). While our analysis revealed no significant association between the Aβ plaque burden and the context fear memory in CRND8 mice at any age of testing, surprisingly, we found positive associations between Aβ plaque burden and tone fear memory at three (r_*S *_= 0.67, *P *< 0.05) and six months of age (r_*S *_= 0.80, *P *< 0.01), but not at 12 months of age (r_*S *_= 0.30, NS). Despite a much reduced sample size, these *post-hoc *analyses revealed that at the ages of three and six months, which are characterized by rapid Aβ plaque formation, those CRND8 mice which showed more Aβ plaques, also showed higher tone fear memory. At present, these preliminary results have to be interpreted with caution. These findings should be replicated in future studies and the relationship between the soluble Aβ and Aβ sequestered in plaques, and also other processes, such as reactive gliosis and inflammatory responses should be systematically evaluated in order to elucidate further the relationship between amyloid-β and cognition at the early stage of plaque formation.

## Discussion

The present results extend previous studies showing that other APP mouse models exhibit impairment in fear conditioned memory, by demonstrating that this impairment is progressive and correlates well with overall Aβ burden. Also, the demonstrated greater sensitivity of the foreground tone conditioning test in the identification of age dependent onset of the memory impairment in CRND8, suggests that this testing paradigm might be particularly suitable in studies evaluating potential therapeutic agents related to memory improvement in APP mouse models.

APP transgenic mouse models have been reported to show memory deficits similar to those observed in AD [21,48-53]. However, comprehensive cognitive profiles, including multiple memory systems, have often been based on comparative analyses from several independent studies using APP mouse models (see [21]). In our study, we simultaneously evaluated two memory systems; memory of the association between the context of the training environment and a foot-shock, which depends on the hippocampus, and memory of the association between a tone and a foot-shock, which is dependent on an intact amygdala. The strength of both types of memory in this paradigm is inferred from the same behavior of freezing response to relevant conditioned stimuli. The implementation of the delay fear conditioning paradigm, in which an explicit cue such as a tone is co-terminated with a foot-shock, usually results in stronger foreground conditioning to tone and weaker conditioning to background contextual cues [54]. Our study confirmed this prediction and demonstrated that nTg control mice had a stronger conditioned tone fear memory than a context fear memory. In contrast, the foreground fear conditioning to tone did not differentiate the response of CRND8 mice from their response to the background context cues. The apparent dissociation in the onset of the cognitive impairment of CRND8 mice in the delay conditioning paradigm has important practical consequences. First, it stresses the importance of the comparative analysis between genotypes across multiple tasks, which differ in the strength of memory development, in order to identify the ceiling performance or maximum dynamic range of the control nTg mice maintained on a specific genetic background. Second, the comparison between the tasks demonstrated that not only the impairment of CRND8 mice declined with age, but they also were not able to reach a level of performance comparable to nTg controls at the earlier ages of testing when the Aβ plaque burden was relatively low. Moreover, the CRND8 mice showed impairment in generalizing the conditioning effects to additional cues present in the testing room, such as characteristics of sound attenuating chambers or other subtle cues, which despite our effort, could not be completely eliminated during the tone test. Consequently, their freezing rates during the pre-tone phase of the test were significantly lower than the freezing of nTg littermates, especially at older ages. Our results also indicated that the 12-month-old nTg control mice showed slightly lower, albeit not significant, freezing rates. Although aged, 19- to 20-month-old, C57BL/6 mice show impairment in the fear conditioning memories [55], additional studies should establish whether the decrease in the fear conditioned freezing response occurs reliably at much earlier ages in the hybrid C57BL/6//C3H background of the CRND8 model. Future studies should also extend our findings and focus on testing the CRND8 mice at ages preceding overt amyloid-β deposition, in an attempt to elucidate whether the impairment in conditioned fear memory in this model contains an age-independent component [56], caused by the constitutive expression of the *APP *transgene. It has been demonstrated that fear memory in another APP Tg2576 mouse model was impaired before the first detection of soluble oligomeric Aβ species [57,58], which seems to support this hypothesis. In summary, our results show that at 12 months of age the CRND8 mice are significantly impaired in both context and cue fear memory, regardless of the salience of the available conditional stimuli, and that the sensitivity of the delay fear conditioning paradigm to identify the onset of impairment depended on the dynamic range of responses shown by control littermates to more salient foreground tone conditioning. The increased salience of the tone conditioned stimulus, which immediately preceded the foot-shock, resulted in greater sensitivity of the detection of memory deficiency in CRND8 mice due to the stronger shift of the nTg mice to the salience of foreground (tone) stimulus. By inference, our results indicate that the compromised hippocampal-amygdala function in CRND8 mice likely impaired the processing and the use of the more salient conditional tone stimulus [59,60]. It is likely, then, that the impairment in the detection of the salience of the foreground (tone) stimulus reflects subtle differences in the learning ability of CRND8 mice at early stages of amyloid pathology.

The comparable context fear memory of the genotypes at three and six months contrasts with the results of our previous studies which demonstrated significant impairment of CRND8 mice in the hippocampus-dependent spatial reference memory evaluated in the water maze test at these ages [26,38]. This discrepancy can be reconciled since the spatial reference memory evaluated in the water or Barnes mazes is not associated with contextual fear memory [61,62] and each of these distinct types of memories might have different underlying mechanisms [63], following different biological functions and adaptive significance. It is also likely that the change in the salience of the conditioning context [64,65] or switching the context conditioning from background to foreground, by eliminating the delay component of tone presentation, might increase the sensitivity of the context testing paradigm in identifying the impairment of the CRND8 mice in this type of memory at earlier ages. The advantage of the fear conditioning testing paradigm lies in its rapid development of robust and long-lasting memory, which is based on an evolutionary anti-predatory fear response preserved across many species, including humans. This paradigm, with its long lasting memory of the CS-US association provides easier implementation of tests focusing on memory acquisition, forgetting and extinction, and it is less physically demanding than the water maze test.

Our study also confirmed the early age of onset [38], followed by rapidly progressing Aβ deposition in CRND8 mice. The deposition of Aβ plaques increased about 12-fold between three- and six- month and four-fold between six- and 12-month-old mice. This increase in Aβ plaque burden was significantly correlated with the decline in contextual and tone fear memory. The importance of these results lies in the validation of the CRND8 model as a research tool which is sensitive to reveal the relationship between Aβ accumulation and cognitive function, with the potential to evaluate the efficacy of pre-clinical therapeutic approaches aiming at improvement of the cognitive function.

There is considerable controversy related to the functional link between the insoluble Aβ residing in plaques and cognitive dysfunction in AD [66-69] or in normal aged individuals [70]. However, the available post mortem evidence indicates significant associations between amyloid pathology and cognition in AD patients [6,71-73], with total amyloid load or burden being the most reliable and powerful manifestation of clinically diagnosed dementia [74]. While Aβ plaque burden does not likely represent the immediate causal factor underlying dementia, our results suggest that it might be a robust surrogate marker indicating the severity of the impairment, at least in the fear conditioning paradigm applied in pre-clinical research using mouse models.

## Conclusions

The advantage of applying the fear conditioning paradigm to evaluate cognitive dysfunction in human studies is that the test focuses on nondeclarative, unconscious memory, which depends on frontal and temporal regions, including cortical sensory processing areas, the thalamus, and the amygdala [75-77]. Several studies demonstrated that in humans fear conditioned memory also depends on the same neural structures that are affected at the early stage of AD [78-81]. Also, unlike declarative or conscious memory, nondeclarative, implicit memory depends less on subjective recall and recognition of information [82,83], providing a better comparative platform between pre-clinical studies involving animal models, and clinical studies of human dementia with neurodegeneration. Although few studies have demonstrated that fear conditioned memory is impaired in AD [84] and in frontotemporal lobar degeneration [85] (of note, an unconditional stimulus used in these studies was a one second burst of 100 db white noise presented through headphones), the association between the impairment in implicit memory and amyloid plaque load in AD patients assessed *in vivo *[86] has yet to be addressed.

## Abbreviations

AD: Alzheimer's disease; Aβ: amyloid beta; ANOVA: analysis of variance; APP: amyloid precursor protein; CR: conditioned response; CS: conditioned stimulus; CSF: cerebrospinal fluid; CV: coefficient of variation; c.p.s: clicks per second; FA: formic acid; FC: fear conditioning; NS: non-significant; nTg: non-transgenic mice; SEM: standard error of the mean; SDS: sodium dodecyl sulfate; Tg: transgenic mice; UR: unconditioned response; US: unconditioned stimulus.

## Competing interests

The authors declare that they have no competing interests.

## Authors' contributions

CJ conceived and supervised the study, analyzed the data and prepared the manuscript. AH and KI prepared the mice and executed the behavioral experiments, collected the data, and performed the evaluation of Aβ burden. PD participated in the Aβ staining. DD designed and carried out the brain sectioning, staining, and preparation of brain slices for the analyses. TG provided the mice and participated in the revision of the manuscript. All authors read and approved the final manuscript.
